# Use of videogames, Internet Gaming Disorder, and Agressiveness in Emerging Adulthood

**DOI:** 10.1192/j.eurpsy.2024.814

**Published:** 2024-08-27

**Authors:** D. Silva, B. R. Maia

**Affiliations:** ^1^ Universidade Católica Portuguesa; ^2^Universidade Católica Portuguesa, Center for Philosophical and Humanistic Studies, Braga, Portugal

## Abstract

**Introduction:**

Internet gaming disorder has been associated with an excessive number of hours spent playing video games, which leads to a detriment of other daily activities and interests. In addition, it is associated with personality traits such as aggression and anger. In Portugal this relations are almost unexplored.

**Objectives:**

To explore the relationship between video game use, internet gaming disorder and aggression.

**Methods:**

This study was applied to a sample of 202 subjects, aged between 18 and 29 years old (M = 22.5, *SD* = 3.006). Subjects fulfilled a sociodemographic and viodeogame pattern questionnaire, and the Portuguese versions of the Internet Gaming Disorder Scale (short form), and the Buss-Perry Aggressiveness Questionnaire.

**Results:**

In this study 20.3% (*n* = 27) of the males and 5.8% (*n* = 4) of the females use excessively videogames, considering the screen time recommended by the American Academy of Pediatrics. However, 81% (*n* = 70) of the sample have a positive self-perception of their use. 21.4% (*n* = 45) use videogames during day, 71% (*n* = 49) during night and 3.8% (*n* = 8) during dawn. The total score for internet gaming disorder was of 15.17 (SD = 6.006), but only 1 subject (0.5%) presented probable videogame disorder considering the sutt-off points. A positive and significant correlation was found between internet gaming disorder and physical agressiveness (.32**), verbal agressiveness (.28**) and hostility (.45**). Finally, a positive correlation was found between internet gaming disorder and time spent playing video games.

**Conclusions:**

Our results, despite being merely exploratory, show us the relationship that exists between the use of video games, internet gaming disorder and aggressiveness. In that sense it is important to continue to explore internet gaming etiology and consequences.

**Disclosure of Interest:**

None Declared

